# Toward Mapping Spatiotemporally Resolved Transcriptomes and RNA‐Protein Interactions

**DOI:** 10.1002/wrna.70057

**Published:** 2026-09-30

**Authors:** Antto J. Norppa, Minna‐Liisa Änkö

**Affiliations:** ^1^ Faculty of Science and Engineering Åbo Akademi University Turku Finland; ^2^ Faculty of Medicine and Health Technology Tampere University Tampere Finland

## Abstract

RNAs navigate highly regulated life cycles that include synthesis, processing, ribonucleoprotein complex formation, localization, and degradation. However, most transcriptome‐wide methods capture static, cell‐wide averages that obscure the spatiotemporal aspects—where and when—of RNA life. Here we review recent advances that bring spatial and temporal resolution to transcriptome and RNA‐protein interaction analyses by labeling RNA either in defined subcellular locales (RNA proximity labeling) or during defined transcriptional time windows to capture the kinetics of RNA synthesis, degradation, and processing (RNA metabolic labeling). We further discuss how RNA labeling approaches can be integrated with RNA‐protein interaction mapping, opening new possibilities for studying ribonucleoprotein dynamics in space and time. Finally, we highlight emerging developments and outline future directions for spatiotemporal analysis of the RNA life cycle.

## Graphical abstract

RNA labeling approaches capture transcriptomes and RNA‐protein interactions in space and time, providing insights into the subcellular organization of gene expression, kinetics of the RNA life cycle, and spatiotemporal remodeling of ribonucleoproteins.

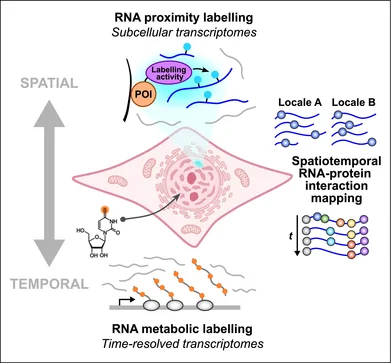

## 1 Introduction

Following synthesis, cellular RNAs undergo a complex life cycle during which they are processed, localized to their site of action, and after performing their function ultimately degraded. RNAs rarely function in isolation, but form complexes with proteins, and sometimes also with other RNAs. These ribonucleoproteins (RNPs) are dynamic assemblies that undergo several compositional changes during the RNA life cycle (Choi et al. 2024; Kilchert et al. 2020; Street et al. 2024). Particularly, in the highly compartmentalized eukaryotic cell, the biogenesis and function of RNPs are often tied to specific subcellular contexts (Biayna and Dumbović 2025; Chekulaeva 2024). Furthermore, between synthesis and degradation, the rates of intermediate life cycle steps such as processing are highly regulated and may account for a significant part of the total RNA lifetime (Ietswaart et al. 2024; Müller et al. 2024; Steinbrecht et al. 2024). Yet, most transcriptome and RNA‐protein interaction analyses reflect cell‐wide, steady‐state averages, obscuring the spatial and temporal dimensions of the RNA life cycle.

Recent methods development has enabled spatiotemporally resolved transcriptomics analyses and, increasingly, RNA‐protein interaction profiling. In this review, we focus on a subset of these technologies: approaches that label RNA in specific subcellular locales (proximity labeling) or RNAs synthesized during a defined labeling period (metabolic labeling) and use sequencing‐based readout to detect the labeled molecules. We discuss the currently available toolkit of RNA proximity and metabolic labeling methods, as well as how these can be combined with RNA‐protein interaction profiling to resolve RNA‐protein interactions in space and time. Finally, we discuss nascent and potential future developments in the field.

## 2 Overview of RNA Labeling Approaches for Spatiotemporal Transcriptomics

A range of methods have been developed that combine in‐cell labeling of RNA with sequencing‐based readout. Here, we focus on proximity labeling (Section 3), which is used to determine the local transcriptomes of subcellular compartments, and metabolic labeling (Section 4) used to capture snapshots of actively transcribed genes as well as to measure kinetics of RNA metabolism. In proximity labeling, a subcellular marker protein is fused to a labeling activity that covalently marks proximal RNAs with a chemical adduct that can be used to identify the transcripts originating from the subcellular locale of interest. In metabolic labeling, cells are fed with synthetic nucleotide precursor analogues that get incorporated into RNA by the cell's transcription machinery.

RNA proximity and metabolic labeling utilize a variety of labeling approaches and chemistries that are discussed in detail in Sections 3 and 4. However, despite differences in the labeling step, these methods discussed converge on just a few basic detection modalities (Figure 1). The first strategy (Figure 1A) uses affinity capture to biochemically enrich the labeled RNAs for short or long‐read RNA sequencing. In the second strategy (Figure 1B), the labeled nucleotides are detected by their effect on reverse transcription, resulting in mutations, deletions, or reverse transcriptase (RT) stops that can be used to classify sequencing reads as labeled or unlabeled. In the third strategy (Figure 1C), the labeling status of individual RNA molecules is interrogated by direct RNA sequencing (dRNA‐seq) through nanopores. Note that these strategies are not necessarily mutually exclusive.

**FIGURE 1:**
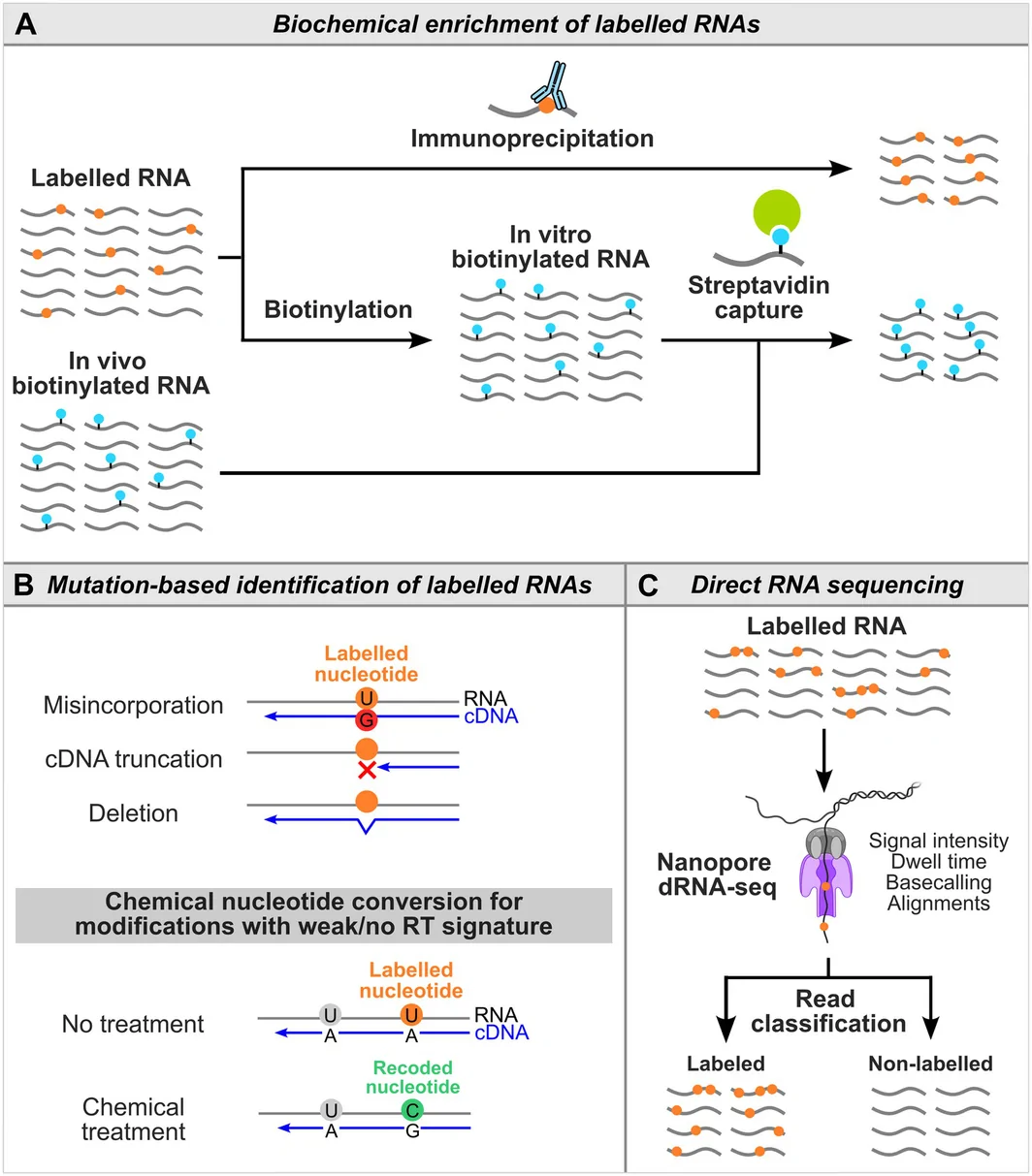
Strategies to capture and detect labeled RNAs in proximity and metabolic labeling experiments. (A) Biochemical enrichment of labeled RNAs. RNA can be biotinylated in vivo, or the initial labeling adducts can be conjugated to biotin in vitro to allow streptavidin‐based capture. Alternatively, labeled RNAs can be enriched by IP using antibodies specific to modified nucleotides. (B) Labeled RNAs can also be identified by detecting reverse transcriptase‐induced mutations in sequencing reads. For some labeling adducts, additional chemical treatments (nucleotide conversion) are required if their effect on reverse transcription is weak. (C) Labeling status of RNAs can also be interrogated by direct RNA sequencing through nanopores.

### 2.1 Biochemical Enrichment of Labeled RNAs

The labeled RNAs in cells can comprise only a minute fraction of the total cellular RNA content. This is particularly the case when very short labeling times are used in metabolic labeling experiments, and in proximity labeling experiments targeting cellular locales with low RNA content. Biochemical enrichment of the labeled RNAs (Figure 1A) enables sequencing effort to be focused on the RNA subpopulation of interest, reducing the required sequencing depth and hence, the cost. However, enrichment steps result in more complicated experimental procedures, can introduce noise and biases in the data and may complicate normalization. In enrichment‐based metabolic labeling experiments, labeled spike‐in RNAs can be added before enrichment to control for variations in recovery and downstream processing, while unlabeled spike‐ins can be used to estimate nonspecific carryover of unlabeled RNA to the labeled RNA fraction (Lugowski et al. 2018; Neymotin et al. 2014; Schwalb et al. 2016). The spike‐in control RNAs may be synthetic transcripts, or labeled and unlabeled RNA from a sufficiently divergent species or two different species (Lugowski et al. 2018). In RNA proximity labeling, spike‐ins are less commonly used. Instead, enrichment at a specific subcellular locale is typically quantified relative to input RNA (Engel et al. 2022), an unlabeled control (Fazal et al. 2019), or another subcellular locale (Padrón et al. 2019). Regardless, in biochemical enrichment‐based methods, no distinction is typically made between labeled RNAs and contaminating unlabeled RNAs at the sequence level. An exception are hybrid detection strategies combining biochemical enrichment with detection of labeling adduct‐induced mutations, which can reduce background coming from unlabeled transcripts (Schofield et al. 2018).

Apart from methods using antibodies for enrichment (e.g., metabolic labeling with five‐bromouridine, BrU, see Section 4.1.3), RNA biotinylation and capture using streptavidin beads is near‐ubiquitously used as the biochemical enrichment method of choice for RNA proximity and metabolic labeling as it enables stringent purification conditions. RNA can either be biotinylated in vivo by a labeling activity as in APEX‐seq (Section 3.1.1), or labeling can introduce an orthogonal chemical handle on the RNA that enables a biotin moiety to be chemically installed in vitro. Most methods discussed here utilize click chemistry for biotin conjugation. Click chemistry refers to a set of easy‐to‐execute, high‐yield, stereoselective chemical reactions widely used for bioconjugation; for details, the reader is referred to recent in‐depth reviews (Fantoni et al. 2021; Venrooij et al. 2024). For RNA applications, the most prominent types of click chemistry are CuAAC (copper‐catalyzed azide–alkyne cycloaddition), SPAAC (strain‐promoted azide–alkyne cycloaddition), and IEDDA (inverse electron demand Diels–Alder reaction). While CuAAC is the most commonly used chemistry due to its high efficiency and small size of terminal alkyne handles, the copper ions in the reaction have been shown to cause RNA fragmentation (Hu et al. 2021). A move toward copper‐free chemistries such as SPAAC and IEDDA will be beneficial, particularly, with wider adoption of long‐read sequencing for detection to preserve full‐length transcripts. Utilization of mutually or sequentially orthogonal click chemistries holds promise for combining multiple RNA labeling methods in the same experiment (Venrooij et al. 2024) (see Section 6).

### 2.2 Mutation‐Based Identification of Labeled RNAs

The sensitivity of RTs to the presence of chemical RNA modifications has long been exploited for mapping of both naturally occurring and experimentally introduced modifications (Motorin and Helm 2024). Upon encountering a modified nucleotide, the RT may arrest, skip, or misincorporate a noncognate nucleotide opposite to the modified nucleotide, manifesting as truncated cDNAs, deletions, or mutations in the sequencing reads, respectively. The “RT signature” of an RNA modification (Figure 1B) is highly dependent on factors such as the enzyme used (Werner et al. 2020), the conditions of the reverse transcription reaction (Kristen et al. 2020), and the sequence context of the modified nucleotide (Zhou et al. 2018). While many RNA modifications have no or only modest effect on reverse transcription under given conditions, altering reaction conditions, using RTs specifically engineered for their detection (Huber et al. 2023), or carrying out a specific chemical or enzymatic pretreatment of RNA (Zhao et al. 2024) may enable RT‐based detection.

In RNA labeling studies, detection of labeling adduct or nucleoside analogue‐induced mutations consistent with their specific RT signatures is used to identify which sequencing reads originate from labeled RNAs (Figure 1B) (Herzog et al. 2017; Lo et al. 2025; Riml et al. 2017; Schofield et al. 2018). While some adducts (e.g., 8‐oxoguanosine, 8‐oxoG, Section 3.1.2) have a strong RT signature, that is, result in high mutation rates without additional treatments, those with weak or no RT signature generally require chemical nucleotide conversion to recode the Watson‐Crick interface of the modified nucleotide and to obtain a strong RT signature that can be utilized for read classification. For instance, 4‐thiouridine (4‐SU, Section 4.1.1.) can be chemically modified to behave as a cytidine in reverse transcription or fully converted to a native cytidine.

### 2.3 Identification of Labeled RNAs by Direct RNA Sequencing

Nanopore sequencing is currently unique in its ability to sequence and detect chemical modifications in native RNA via dRNA‐seq. In nanopore sequencing (reviewed by Meyer et al. 2026; Wang et al. 2021), an engineered transmembrane protein is embedded into a membrane across which a voltage is applied, generating an ionic current. As a nucleic acid strand is translocated through the pore at a rate controlled by a helicase, the current changes in a manner characteristic of the specific stretch of nucleotides occupying the pore. The raw current‐over‐time output is interpreted by a basecalling algorithm to infer the nucleotide sequence for each read. Importantly, the ionic current also carries information on nucleotide modifications, which can cause alterations in raw signal features including signal intensity and dwell time (i.e., the time that a nucleotide resides in the nanopore), as well as errors in basecalling. Modified nucleotides can therefore be identified computationally based on raw signal features, basecalling errors, or a combination of both following standard basecalling (reviewed by Furlan, Delgado‐Tejedor, et al. 2021; Wan et al. 2022). Alternatively, modification detection can be integrated directly into the basecalling step (modification‐aware basecalling) (Cruciani and Novoa 2026). As a readout for RNA labeling experiments, dRNA‐seq enables computational classification of molecules as unlabeled and labeled with relatively minimal prior manipulations (Figure 1C) (Maier et al. 2020; Martinek et al. 2024).

## 3 RNA Proximity Labeling: From Global to Local Transcriptomes

RNA proximity labeling encompasses methods that use localized enzymatic or chemical reactions to label RNAs within a specific spatial neighborhood. A protein marker localizing to the subcellular structure of interest is fused to a labeling activity that generates radicals or other short‐lived diffusible chemical species. The resulting cloud of reactive molecules rapidly labels proximal RNAs, with the probability of labeling decreasing with distance from the labeling activity. The spatial resolution of proximity labeling is dependent on the labeling chemistry, including the lifetime and diffusion coefficient of the reactive chemical species, the experimental conditions, as well as the subcellular environment, by providing differing levels of quenching and barriers for diffusion (Sen and St Johnston 2026; Skovsen et al. 2005). Although labeling can extend beyond the boundaries of the subcellular locale of interest, proximity labeling methods have been successful in enriching distinct transcriptomes even for adjacent, nonmembrane‐separated domains. Proximity labeling methods also possess high temporal resolution, as sufficient labeling can be achieved in under a minute to tens of minutes depending on the method. This has enabled studies of subcellular transcriptome dynamics in response to external cues as well as the combination of metabolic and proximity labeling to track newly transcribed RNAs (Fazal et al. 2019; Li et al. 2022). After labeling, current methods either utilize chemical handles installed on modified nucleotides for biochemical enrichment or identify labeled RNAs from sequencing reads without enrichment by detecting RT mutations induced by the labeling adducts (Figure 1A,B).

We first review the methodology of RNA proximity labeling (Section 3.1) and then highlight the biology these methods can reveal by discussing proximity labeling studies of RNA granules (Section 3.2).

### 3.1 RNA Proximity Labeling Approaches

Current RNA proximity labeling methods can be divided into two main categories based on the mechanism of proximity labeling. Enzymatic proximity labeling methods utilize local enzyme‐catalyzed generation of reactive chemical species that label proximal RNAs. In contrast, photocatalytic proximity labeling methods are based on local light‐induced labeling of proximal RNAs through oxidative or nonoxidative mechanisms. We provide a summary of the various proximity labeling methods discussed in this review in Table 1.

**TABLE 1:** Summary of RNA proximity labeling methods.

Method	Labeling mechanism	Detection modality	Genetic fusion	Reactive species	Experimental notes
APEX‐seq	Enzymatic	Enrichment	APEX2	Phenoxyl radical	+ Reagents readily available
					+ Alternative substrates and approaches
					− Long radical lifetime, no saturation of labeling
					− Permeability issues in some cell types
BAP‐seq	Enzymatic	Enrichment	BS2 esterase	Acylating agent	+ Local enzymatic activation of reactive species
					+ BS2 plasmids available
					− Probe may need to be synthesized
CAP‐seq	Photocatalytic, oxidative	Enrichment	miniSOG	Singlet oxygen	+ Local enzymatic generation of singlet oxygen
					+ miniSOG plasmids available, no need for chemical probe
Halo‐seq	Photocatalytic, oxidative	Enrichment	HaloTag	Singlet oxygen	+ Versatile HaloTag useful also for other applications
					− Halo‐DBF probe may need to be synthesized
FAP‐seq	Photocatalytic, oxidative	Enrichment	FAP	Singlet oxygen	+ MG‐HI probe locally activated by FAP binding
					− MG‐HI probe may need to be synthesized
SeNB‐based labeling	Photocatalytic, oxidative	Enrichment	—	Singlet oxygen	+ Fusion protein expression not required
					− Restricted to organelle membranes
					− SeNB derivatives may need to be synthesized
OINC‐seq	Photocatalytic, oxidative	Mutation	HaloTag	Singlet oxygen	+ Streamlined protocol with no enrichment steps
					+ Versatile HaloTag useful also for other applications
					− Halo‐DBF probe may need to be synthesized
μMap‐seq	Photocatalytic, nonoxidative	Enrichment	HaloTag	Carbene	+ Low nanometer resolution
					+ Versatile HaloTag useful also for other applications
					− Iridium photocatalyst may need to be synthesized

#### 3.1.1 Enzymatic RNA Proximity Labeling

The first category of RNA proximity labeling methods utilizes enzymatic activities to generate reactive chemical species for RNA labeling. APEX‐seq is based on genetic fusion of an engineered ascorbate peroxidase, APEX2, with a protein marker localizing to the subcellular compartment of interest (Fazal et al. 2019). Cells expressing the APEX2‐fusion are treated with biotin–phenol, followed by a brief pulse (1 min) of hydrogen peroxide (H_2_O_2_) to induce APEX2‐catalyzed one‐electron oxidation of biotin–phenol to a biotin–phenoxyl radical (Figure 2A,B). The phenoxyl radicals react with proximal RNAs primarily at guanosine residues, which have the lowest oxidation potential among the standard RNA nucleobases, forming an adduct at the C8 position (Fazal et al. 2019; Wilbanks et al. 2021) (Figure 2A). The in‐cell biotinylated RNAs are enriched from total RNA using streptavidin beads and sequenced.

**FIGURE 2:**
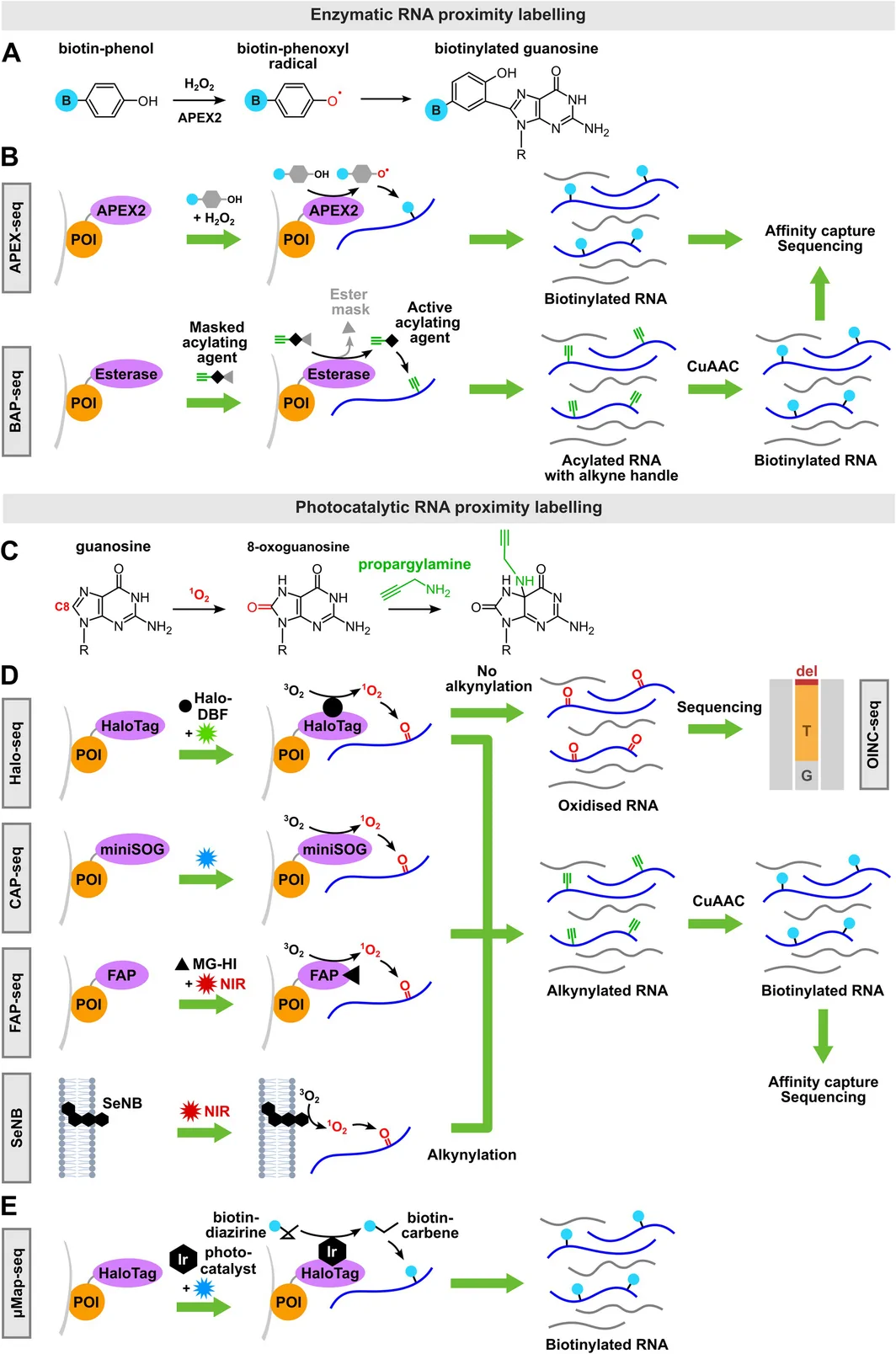
RNA proximity labeling methods. (A, B) Enzymatic proximity labeling methods utilize enzymatic activities that generate reactive chemical species to label proximal RNAs. (A) Generation of biotin‐phenoxyl radicals and biotinylation of guanosines in APEX‐seq. (B) Schematic view of APEX‐seq and BAP‐seq. (C, E) Photocatalytic proximity labeling methods are based on light‐induced generation of reactive chemical species that label proximal RNAs through oxidative or nonoxidative mechanisms. (C) In oxidative photocatalytic proximity labeling, guanosines are oxidized to 8‐oxoguanosines, which can be alkynylated to enable subsequent click reactions. (D) Schematic view of oxidative photocatalytic proximity labeling methods. (E) Schematic view of μMap‐seq, a nonoxidative photocatalytic proximity labeling method.

The labeling radius of APEX2 is commonly approximated at ~20 nm for proteins (Rhee et al. 2013). Recent nanoruler‐based measurements show strong contact‐dependent labeling at < 10 nm and weaker radical‐based labeling at longer distances for phenol substrates (Yang et al. 2025). Phenoxyl radicals have a lifetime in the low‐millisecond range, which is relatively long compared to the lifetimes of other reactive species used in proximity labeling (nanoseconds to microseconds, see below). Consequently, the labeling range appears to be limited by diffusion rather than radical lifetime, and saturation of proximate reactive targets can lead to spreading of labeling over multiple micrometers, underlining the importance of optimized labeling conditions (Sen and St Johnston 2026). Other limitations of the APEX‐seq method include the relatively low reactivity of biotin−phenol, the limited biotin permeability of some cell types such as human induced pluripotent stem cells (iPSCs) (Tschan et al. 2025), as well as the reliance on fusion protein expression. To improve sensitivity, several studies have explored the use of alternative substrates such as biotin−aniline (Zhou et al. 2019) and smaller, biotin‐free substrates carrying an alkyne or azide group that enables biotin conjugation via click chemistry (Li et al. 2020; Liang et al. 2024). Furthermore, metabolic incorporation of the electron‐rich nucleoside analogues 4‐SU and 6‐thioguanosine (6‐SG, Section 4.1.1) prior to APEX2‐mediated labeling was shown to increase the sensitivity of APEX‐seq due to their higher reactivity with the electrophilic phenoxyl radicals (Huang et al. 2021; Li et al. 2022). Consequently, with the relatively short metabolic labeling time utilized, labeling became strongly biased toward newly synthesized transcripts, no longer reflecting steady‐state RNA distribution. Extending the metabolic labeling time could be used to reduce this bias when a more evenly labeled RNA pool is desirable. Conversely, the nascent RNA bias could be a useful feature for distinguishing transiently expressed transcript from more stably associated ones. The requirement for APEX2 fusion protein expression can be bypassed by carrying out the proximity biotinylation in fixed cells through antibody‐ or hybridization‐based targeting of APEX2 to the cellular location of interest (Choudhury et al. 2024; Yap et al. 2025).

Electrophilic molecules that selectively acylate the 2′‐hydroxyl group of ribose are widely used for RNA structure probing and other applications (Shioi and Kool 2024). Recently, Pani et al. developed BAP‐seq (bioorthogonal acylating agents for proximity labeling and sequencing), which utilizes novel “masked” acylating agents that enter the cell as inert precursors and are therein enzymatically activated (Figure 2B) (Pani et al. 2024). To harness this concept for proximity labeling, an esterase from *Bacillus subtilis* is genetically fused to a subcellular marker protein and the cells are supplied with an acylating agent carrying an ester‐based masking group. The esterase hydrolyses the masking group to form an enol which undergoes rapid enol–keto tautomerization, giving rise to a highly reactive acylating species. An alkyne or azide handle allows subsequent biotin conjugation via click reactions and affinity capture using streptavidin beads. Although alternative detection modalities are yet to be explored, by analogy, acyl adducts formed by SHAPE (selective 2′‐hydroxyl acylation analyzed by primer extension) reagents used in RNA structure probing have been detected as RT stops, RT mutations, and base‐calling errors during Nanopore (dRNA‐seq) (reviewed by Xiao et al. 2024).

#### 3.1.2 Photocatalytic RNA Proximity Labeling

The second category of RNA proximity labeling methods is based on localized light‐activated generation of reactive chemical species that label nearby RNAs (Figure 2C−E). Most methods in this category involve generation of reactive oxygen species (ROS) through diverse mechanisms (Figure 2D), based on either small molecule or genetically encoded photosensitizers (Engel et al. 2022; Li, Han, et al. 2024; Lo et al. 2025; Wang et al. 2019). In CAP‐seq (chromophore‐assisted proximity labeling and sequencing), ROS are generated by miniSOG, a fluorescent flavoprotein engineered from an *Arabidopsis thaliana* blue light photoreceptor (Wang et al. 2019). Fusing miniSOG to a protein of interest (POI) enables local ROS production upon blue light illumination. Recently, a miniSOG variant called Lantern was developed through directed evolution, resulting in greatly improved labeling efficiency enabling subminute labeling times (Fang et al. 2026). In Halo‐seq and its derivative OINC‐seq (oxidation‐induced nucleotide conversion sequencing) (Engel et al. 2022; Lo et al. 2025), ROS originate from a small molecule fluorophore, dibromofluorescein (DBF). Spatial specificity is achieved by genetically fusing the POI to a HaloTag, a self‐labeling protein tag. Upon addition of Halo‐DBF to cells expressing a POI–HaloTag fusion, it becomes covalently attached to the HaloTag. Excess ligand is then washed away and ROS generation induced by green light illumination. Since Halo‐DBF produces ROS upon illumination whether or not it is HaloTag‐bound, low‐level background RNA oxidation can occur in unintended cell locales from the residual ligand. FAP‐seq addresses this limitation by using a malachite green‐based photosensitiser (MG‐HI) that is activated by near‐infrared (NIR) light only when bound to a fluorogen activating protein (FAP) fused to the POI (Li, Han, et al. 2024).

While the previous approaches all rely on fusion proteins for subcellular specificity, a recent study introduced several derivatives of a small molecule photosensitiser, selenium‐containing Nile Blue (SeNB), that preferentially accumulate to distinct organelle membranes without need for protein partners for targeting (Jia et al. 2025). Depending on the specific “tail” attached to a common scaffold structure, the photosensitiser can be targeted to the endoplasmic reticulum, mitochondrial, or lysosomal membranes, and local ROS generation induced by NIR light.

The primary ROS produced by these approaches is a singlet oxygen (^1^O_2_). Reported cellular ^1^O_2_ lifetime values of ~0.6 to 3 μs suggest that most molecules will be quenched or react within roughly 100–300 nm from the source, assuming two lifetimes (Hatz et al. 2007; Moan 1990; Skovsen et al. 2005). Like phenoxyl radicals, ^1^O_2_ preferentially reacts with guanosine at the C8 position, forming 8‐oxoG, which can undergo further oxidation (Lo et al. 2025) (Figure 2C). RNAs containing 8‐oxoG can be alkynylated in living cells, enabling installation of a chemical handle for purification, or 8‐oxoG sites can be detected as RT‐induced mutations. CAP‐, Halo‐, and FAP‐seq converge on the former strategy (Engel et al. 2022; Li, Han, et al. 2024; Wang et al. 2019). Visible or NIR light irradiation is carried out in the presence of propargylamine, a nucleophile that alkynylates 8‐oxoGs. The alkyne group enables conjugation of biotin to labeled RNAs via CuAAC click chemistry and subsequent biotin‐streptavidin affinity purification. OINC‐seq adopts a more streamlined approach, detecting G>T/C mutations or deletions induced by oxidized guanosine adducts using total RNA as input (Lo et al. 2025). Omitting the enrichment steps may reduce experimental noise, while filtering based on modification frequency and the presence of multiple modified nucleotides in the same transcript can help to discriminate between specific labeling and background. However, the sequencing depth required for reliable and sensitive detection of oxidation events in total RNA may make this approach unfeasible for less RNA‐rich cell locales.

Finally, μMap‐seq is a photocatalytic proximity labeling approach that uses a nonoxidative labeling mechanism (Figure 2E) (Knutson et al. 2025). Similar to Halo‐seq and OINC‐seq, μMap‐seq uses a HaloTag‐fused subcellular marker to achieve spatial specificity but conjugates an iridium photocatalyst to HaloTag. Blue light illumination induces energy transfer from the photocatalyst to biotin‐diazirine, leading to formation of reactive carbene species that biotinylate nearby RNAs. Due to the extremely short lifetime of carbenes in water (in the low‐nanosecond range), the μMap method can achieve a spatial resolution of < 10 nm for protein proximity labeling (Bartholow et al. 2023; Oakley et al. 2022). In contrast to oxidative methods, all four nucleotides can be labeled, although the carbenes show modest bias toward G and U (Knutson et al. 2025).

### 3.2 Insights Into the Biology of RNA Granules From Proximity Labeling

In addition to membrane‐bound organelles, eukaryotic cells contain a large number of compositionally and functionally distinct membraneless RNA granules which are formed through biomolecular condensation (extensively covered by recent reviews, see Hirose et al. 2023; Li, Liu, et al. 2024; Ripin and Parker 2023). While some RNA granules are stable enough to be isolated via biochemical means (Shichino and Iwasaki 2025), the degree to which the granules retain their native state during lengthy isolation procedures is difficult to evaluate. As an alternative approach for analyzing RNA granule composition, proximity labeling has proven to be generally applicable. In the nucleus, RNA proximity labeling has been used to determine the RNA composition of nucleoli (Barutcu et al. 2022; Engel et al. 2022; Fazal et al. 2019; Knutson et al. 2025), nuclear speckles (Barutcu et al. 2022; McIntyre et al. 2025), paraspeckles (Knutson et al. 2025), Cajal bodies (Barutcu et al. 2022; Knutson et al. 2025), PML bodies (Barutcu et al. 2022; Knutson et al. 2025), Sam68 bodies (Barutcu et al. 2022), and histone locus bodies (Barutcu et al. 2022). In the cytoplasm, RNA proximity labeling has enabled mapping of stress granule (O'Connell et al. 2026; Padrón et al. 2019; Ren, Tang, et al. 2023) and processing body (P‐body) (O'Connell et al. 2026) transcriptomes.

As a case‐in‐point, recent methodological advances in studying subcellular transcriptomes and nuclear architecture have spurred significant new advances in nuclear speckle biology (Chaturvedi and Belmont 2024). Speckles have long been studied in connection to RNA splicing since the early observations of various spliceosome components localizing to these structures. However, they are now thought to have multifaceted roles as nuclear hubs coordinating transcription, RNA processing, nuclear export, genome organization, and cellular stress responses (reviewed by Chaturvedi and Belmont 2024). While a large number of speckle proteins have been known for decades (Fong et al. 2013; Mintz et al. 1999; Saitoh et al. 2004), with new ones continuing to be identified (Dopie et al. 2020), relatively little has been known about the speckle transcriptome until recent analyses by RNA proximity labeling and other approaches (Barutcu et al. 2022; Khyzha et al. 2025; McIntyre et al. 2025; Wu et al. 2024). These studies show that speckles contain high levels of transcripts with unspliced introns, which can originate from speckle‐adjacent transcription sites or migrate posttranscriptionally to speckles, and include both transiently and more stably associated transcript populations. In particular, speckles are enriched in Barutcu et al. (2022), McIntyre et al. (2025), and Wu et al. (2024) and important for the correct splicing of GC‐rich transcripts with short introns (Małszycki et al. 2026; Tammer et al. 2022). The speckle−nucleoplasm interface is thought to act as a stimulatory environment for transcription (Alexander et al. 2021, 2025; Kim et al. 2019) and RNA splicing (Bhat et al. 2024; Wu et al. 2024), as speckle proximity boosts gene expression levels and increases splicing efficiency. Most recently, speckles were also shown to play a key role in pre‐mRNA cleavage and polyadenylation (CPA) (Yoon et al. 2025). Here, reanalysis of a large APEX‐seq dataset covering nine nuclear locations (Barutcu et al. 2022) showed that uncleaved pre‐mRNAs are highly enriched at speckles, with CPA of > 50% of human genes estimated to take place in association with speckles (Yoon et al. 2025). Thus, fundamental new insights into the spatial organization of splicing and CPA in the nucleus have been gained in recent years.

## 4 RNA Metabolic Labeling: From Static Snapshots to Time‐Resolved Transcriptomes

RNA metabolic labeling encompasses methods where cells are supplied with modified RNA metabolic precursors that are incorporated as nucleotides into newly synthesized RNA (Figure 3). Subsequently, RNAs that were transcribed during the labeling period can be distinguished from pre‐existing RNAs by the presence of these modified nucleotides. While the most direct path for labeling nascent RNA would be supplying cells with modified nucleoside triphosphates (NTPs), NTPs cannot be used for metabolic labeling in intact cells as they do not appreciably cross the cell membrane due to their highly negative charge and lack of transporters. Instead, nucleoside or, less commonly, nucleobase analogues are used, which enter the cell through specific transporters, and are converted to NTPs through de novo synthesis or nucleotide salvage pathways. NTPs are then cotranscriptionally incorporated into nascent RNAs by RNA polymerases.

**FIGURE 3:**
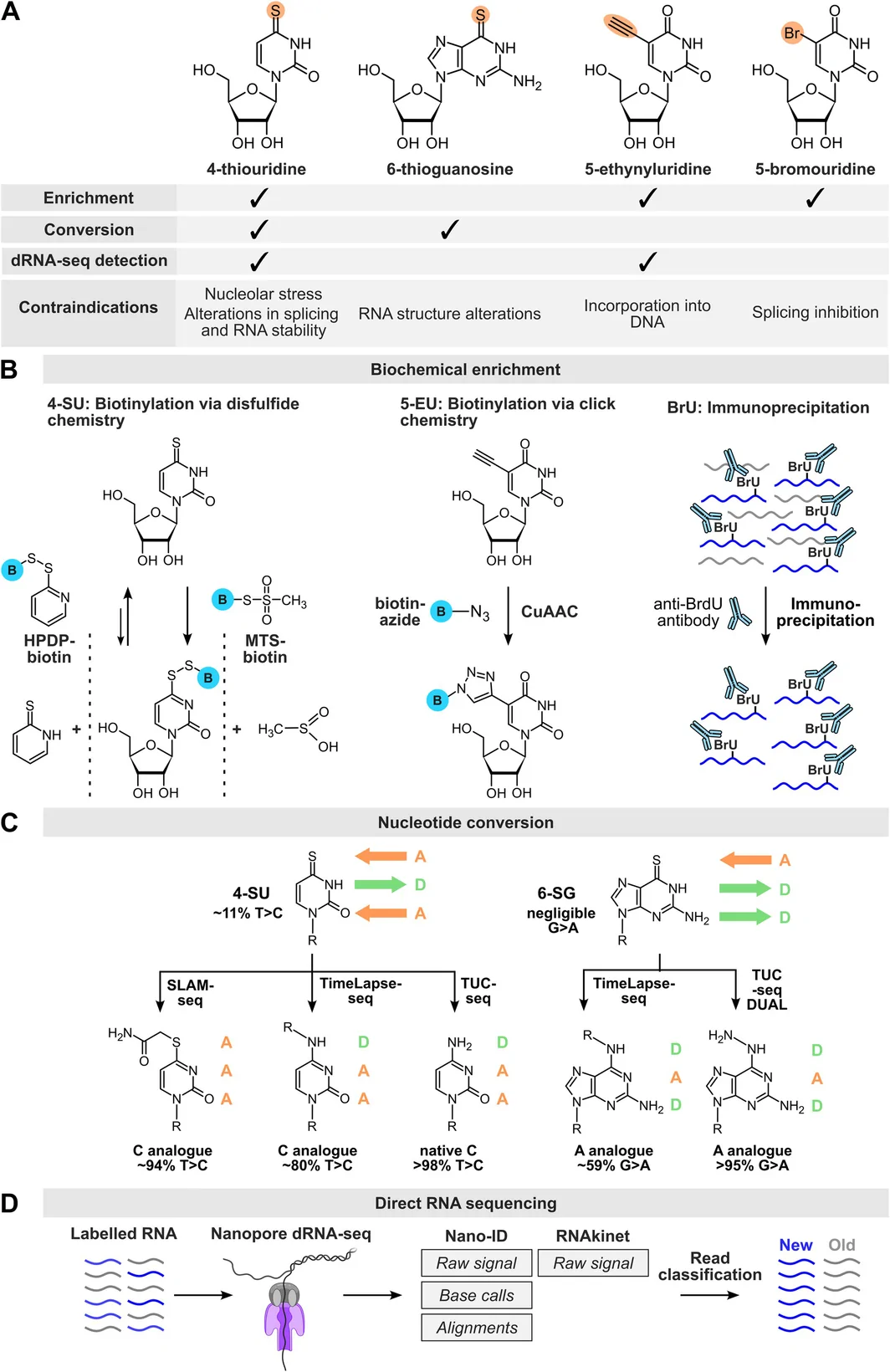
Metabolic labeling using nucleoside analogues. (A) Structures of the nucleoside analogues discussed in this review and their compatibility with various detection modalities, and potential contraindications. (B) Methods for biochemical enrichment of 4‐SU (left), 5‐EU (center), and BrU‐labeled RNAs (right). While 4‐SU and 5‐EU utilize specific chemistries for biotinylation of the nucleoside analogues to allow streptavidin‐based capture, BrU‐labeled RNAs are enriched by immunoprecipitation. (C) Nucleotide conversion approaches for 4‐SU and 6‐SG use chemical treatments to recode the Watson‐Crick interface of the nucleoside analogues to resemble or match those of cytidine and adenosine, respectively. (A) and (D) indicate hydrogen bond acceptors and donors. (D) For 4‐SU and 5‐EU, bioinformatics methods such as Nano‐ID and RNAkinet can be used to classify nanopore dRNA‐seq reads as labeled and unlabeled.

A large variety of nucleoside and nucleobase analogues have been successfully used for metabolic labeling, but we will focus here on nucleosides that are most widely used for temporal gene expression studies in eukaryotic cells (Section 4.1). We will then describe how metabolic labeling can provide insights into the kinetics of the RNA life cycle, including RNA synthesis, processing, and degradation (Section 4.2).

### 4.1 RNA Metabolic Labeling Using Modified Nucleosides

We review here the most commonly used nucleoside analogues. While same types of kinetic experiments (see Section 4.2) can be performed with each of them, the nucleosides differ in their compatibility with the detection modalities discussed in Section 2 (Figure 3A). Another major consideration in the nucleoside choice is the potential side effects of the nucleoside analogues in the biological system under study. Four‐thiouridine has been reported to cause inhibition of ribosomal RNA synthesis and processing (Burger et al. 2013), as well as RNA splicing alterations and increased RNA stability at high incorporation levels (Altieri and Hertel 2021). Six‐thioguanosine was shown to exert position‐specific effects on the thermodynamic stability of RNA duplexes (Gładysz et al. 2019), which could alter RNA function and decay. On the other hand, five‐ethynyluridine (5‐EU) is predominantly incorporated into DNA rather than RNA in some animals, excluding its use in such species (Kjosavik et al. 2025). Thus, phylogenetic differences in nucleotide metabolism warrant caution when adapting metabolic labeling to new biological systems. For sensitive cell types and longer labeling times, BrU may be a preferred choice as it is well‐tolerated even at high concentrations (Tani et al. 2012). BrU was reported to inhibit splicing in in vitro splicing assays when incorporated at levels unlikely to be reached in metabolic labeling experiments (Wansink et al. 1994); however, its impact on splicing in living cells remains to be investigated. Taken together, while various contraindications have emerged from studies focused on individual analogues, systematic comparisons of cellular toxicity, stress responses, and the impact of nucleoside incorporation on RNA structure, stability, and processing are currently lacking.

#### 4.1.1 4‐Thiouridine and 6‐Thioguanosine

Four‐thiouridine and 6‐SG are sulfur‐modified nucleoside analogues in which a carbonyl (C═O) in the uracil or guanine nucleobase is substituted by a thione (C═S) (Figure 3A), giving the analogues chemical reactivity distinct from the respective canonical nucleosides. Four‐thiouridine is compatible with all three detection modalities described in Section 2, making it the most versatile of the nucleosides discussed here. Current methods for biochemical enrichment of 4‐SU‐labeled RNA are based on activated disulfide reagents which enable specific conjugation of biotin to 4‐SU residues (Figure 3B, left). Biotinylated RNAs are captured using streptavidin and eluted by reducing the disulfide bond. HPDP‐biotin (*N*‐[6‐(biotinamido)hexyl]‐3′‐(2′‐pyridyldithio)propionamide) and methane thiosulfonate (MTS)‐biotin are the most commonly used disulfide reagents. HPDP‐biotin suffers from low efficiency, leading to a bias toward longer RNAs (Duffy et al. 2015). In contrast, MTS chemistry gives nearly quantitative reaction yield, shows less length bias, and enables both biotin conjugation via MTS‐biotin or direct capture of 4‐SU‐containing RNAs using MTS‐coupled beads (Duffy et al. 2015, 2018; Sugio et al. 2023). With biochemical enrichment, very short labeling times can be used, particularly if RNA is fragmented before enrichment to avoid sequencing of long, unlabeled 5′ regions of nascent transcripts, an approach introduced in TT‐seq (transient transcriptome sequencing) (Schwalb et al. 2016).

Although 4‐SU leads to a low level of T‐to‐C mutations in sequencing reads (Herzog et al. 2017), chemical nucleotide conversion can be used to greatly increase the mutation frequency. Three distinct chemistries for nucleotide conversion, utilized in SLAM‐seq (thiol(SH)‐linked alkylation for the metabolic sequencing of RNA) (Herzog et al. 2017), TimeLapse‐seq (Schofield et al. 2018), and TUC‐seq (thiouridine‐to‐cytidine‐sequencing) (Lusser et al. 2020; Riml et al. 2017), have been developed for 4‐SU (Figure 3C, left). While SLAM‐seq is based on the alkylation of 4‐SU to a cytidine analogue, in TimeLapse‐seq and TUC‐seq 4‐SU is oxidized to a convertible intermediate that yields a cytidine analogue (TimeLapse‐seq) or a native cytidine (TUC‐seq) upon reaction with an alkylamine or ammonia, respectively. TimeLapse‐seq and modified TUC‐seq chemistries have also been applied to 6‐SG‐labeled RNA (Figure 3C, right), in which case the nucleotide conversion increases the frequency of G‐to‐A mutations that otherwise occur at low level at 6‐SG sites (Gasser et al. 2020; Kiefer et al. 2018). The distinct mutational signatures can be harnessed for sequential 6‐SG/4‐SU labeling (TUC‐seq DUAL), which allows simultaneous determination of synthesis and degradation rates based on the increase in T‐to‐C mutations and decrease in G‐to‐A to mutations, respectively (Gasser et al. 2020) (see Section 4.2.1). This approach has also been adapted for use in single cells (Wang et al. 2025).

In addition to biochemical enrichment and nucleotide recoding, separation of 4‐SU‐labeled and unlabeled molecules can be achieved computationally from Nanopore dRNA‐seq data (Figure 3D) (Coscujuela Tarrero et al. 2024; Maier et al. 2020). In a recent study (Coscujuela Tarrero et al. 2024), a neural network (nano‐ID) was trained using unlabeled RNA and 4‐SU‐labeled RNA isolated by biotin‐streptavidin pulldown and used to estimate a modification probability for each read in experimental samples. Based on the modification probability, reads were then classified as nascent or pre‐existing.

#### 4.1.2 5‐EU

5‐EU is a uridine analogue with an ethynyl group (–C≡CH) linked to the uracil base (Jao and Salic 2008). As a terminal alkyne, the ethynyl group provides access to CuAAC click chemistry for biotin conjugation via biotin‐azide (Figure 3B, center), or visualization of labeled RNAs via azide‐linked fluorophores. Click chemistry‐based biochemical enrichment of 5‐EU‐labeled RNA has been adapted for next‐generation sequencing in the EU‐RNA‐seq method, which uses a pulse of 5‐EU to capture a snapshot of nascent transcriptome (Palozola et al. 2017, 2021). Single‐cell EU‐seq measures synthesis and degradation rates in single cells with 5‐EU pulse‐chase experiments (Section 4.2.1). Here, the biotin click reaction is performed in situ, followed by single‐cell sorting and barcoding, and biochemical separation and sequencing of the labeled and unlabeled RNA fractions (Battich et al. 2020).

Two machine learning tools, nano‐ID and RNAkinet, have been developed for classification of Nanopore dRNA‐seq reads as 5‐EU‐labeled and unlabeled (Figure 3D) (Maier et al. 2020; Martinek et al. 2024). While nano‐ID (also later used for 4‐SU, see above) utilizes the raw electrical signal as well as base‐calling and reference sequence alignment information, RNAkinet identifies 5‐EU‐labeled molecules based on the electrical signal alone. While inferring labeling status of each sequenced molecule from nanopore signal promises to significantly streamline RNA labeling experiments, these approaches currently have limitations related to the nanopore sequencing platform and machine learning. The relatively low throughput of dRNA‐seq compared to short‐read sequencing is limiting the analysis of low‐abundance RNA species. Furthermore, machine learning classifiers may be sensitive to differences between training and application data, including biological factors such as species and cell type, as well as technical factors such as sequencing chemistry and platform. However, RNAkinet demonstrated improved generalization compared to nano‐ID across several tested cell types and organisms (Martinek et al. 2024).

#### 4.1.3 5‐Bromouridine

Five‐bromouridine is a uridine analogue with a bromine substitution at position 5 of the uracil ring. Unlike the sulfur‐substituted nucleosides and 5‐EU, metabolic labeling with BrU does not provide access to specific covalent conjugation or nucleotide recoding chemistries. Instead, BrU‐labeled RNAs are biochemically enriched by immunoprecipitation (IP) with anti‐BrdU (5‐bromodeoxyuridine) antibodies that also recognize BrU in RNA (Figure 3B, right) (Haider et al. 1997). While IP‐based enrichment comes with caveats such as lot‐to‐lot variability in antibodies, the one‐step capture allows streamlined protocols that can achieve high sensitivity and low background (Huang et al. 2026). Next‐generation sequencing methods utilizing BrU labeling and IP‐based enrichment include Bru‐seq, its variant BruChase‐seq (Huang et al. 2026; Paulsen et al. 2013, 2014), and BRIC‐seq (5‐bromouridine IP chase sequencing) (Tani et al. 2012). Bru‐seq utilizes a single time‐point pulse to quantify nascent transcription, while BRIC‐seq and BruChase‐seq use BrU pulse‐chase experiments to measure RNA degradation rates. BrU has also been used in sequential labeling experiments with 4‐SU to measure synthesis and degradation rates, with BrU RNAs captured by IP and 4‐SU RNAs identified through nucleotide conversion (Kawata et al. 2020). Finally, while Nanopore dRNA‐seq detection of BrU has been explored with synthetic RNAs (Fleming et al. 2024; Maier et al. 2020), its feasibility for use with actual BrU metabolic labeling data is yet to be demonstrated.

### 4.2 Using Metabolic Labeling to Study the Kinetics of RNA Metabolism

While total RNA‐seq is routinely used to quantify transcript abundances, most RNA‐seq experiments do not distinguish between the contributions of RNA synthesis and degradation. A high transcript abundance can result from high transcriptional activity or high RNA stability, while low abundance can indicate either low transcriptional activity or rapid degradation. Both mechanisms of maintaining cellular homeostasis are widely utilized. Furthermore, regulation at the level of RNA processing and localization is a significant contributor to gene expression landscapes. Understanding the kinetics of each of these processes can yield insights into the mechanisms of gene regulation.

Even without metabolic labeling, kinetic information can be inferred from total RNA‐seq data. This is typically achieved either through models that rely on significant, often unjustified assumptions, such as constant RNA processing rates or neglecting RNA processing altogether, or through time‐course experiments in which cells are sampled at multiple time points after a perturbation that elicits a transcriptional response (reviewed by Furlan, de Pretis, and Pelizzola 2021). However, by distinguishing newly synthesized RNAs from pre‐existing RNAs, metabolic labeling can more accurately measure the kinetics of RNA synthesis, processing, and degradation.

#### 4.2.1 Kinetics of RNA Synthesis and Degradation

A few basic types of experimental designs are commonly employed to measure the kinetics of RNA metabolism using metabolic labeling (Figure 4). To obtain kinetic information from metabolic labeling data, the measured abundances of labeled and unlabeled RNA are interpreted using kinetic models that describe the synthesis, processing, and degradation of RNA species (for detailed discussion, see Furlan, de Pretis, and Pelizzola 2021; Uvarovskii et al. 2019). Depending on experimental design, rate parameters may be obtained analytically from single time point data or estimated by fitting kinetic models to time‐course data. A *pulse* experiment is the simplest type of metabolic labeling experiment, where a nucleoside analogue is added to the cell medium for a specific time interval before cells are harvested. While a single time‐point pulse experiment can be used to infer rates of RNA synthesis, degradation, and processing, or a subset of these (de Pretis et al. 2015; Hersch et al. 2022; Jürges et al. 2018; Schwalb et al. 2012; Uvarovskii and Dieterich 2017), this inference generally relies on simplifying assumptions which vary depending on the specific computational method. Commonly, RNA metabolism is assumed to be at steady state and synthesis, processing, and degradation are assumed to follow simple first‐order kinetics. The steady‐state assumption may be violated during rapid cellular responses, while the assumption of first‐order kinetics may not be valid, for example, for RNAs with complex decay mechanisms (Deneke et al. 2013).

**FIGURE 4:**
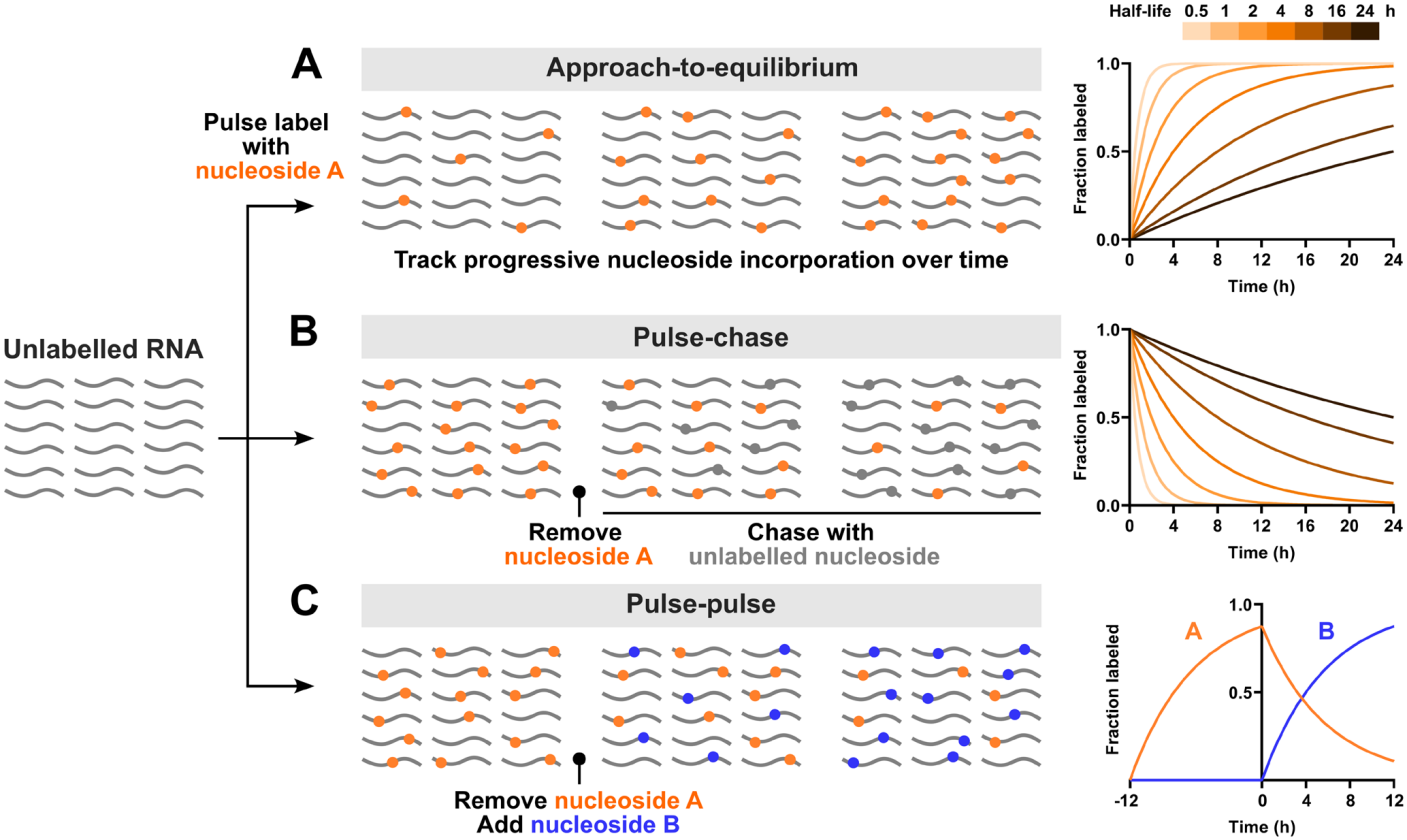
Approaches to study kinetics of gene expression by RNA metabolic labeling. (A) In approach‐to‐equilibrium experiments, a nucleoside analogue is introduced, and cells are sampled at various time points after initiation of labeling. The kinetics of labeled RNA accumulation are related to RNA half‐life, as shown on the right for RNAs with half‐lives ranging from 0.5 to 24 h. (B) In pulse‐chase experiments, cells are pulse‐labeled with a nucleoside analogue, after which the nucleoside is withdrawn, and the corresponding unlabelled nucleoside is introduced. Theoretical decay curves of RNAs with half‐lives ranging from 0.5 to 24 h are shown on the right. (C) In pulse‐pulse experiments, cells are first pulse‐labeled with nucleoside A, after which nucleoside A is withdrawn, nucleoside B is introduced and cells are sampled at different time points. In the example on the right, cells are labeled for 12 h with nucleoside A, followed by 12 h labeling with nucleoside B.

In global studies where thousands of RNA species with a broad range of kinetic rates are studied simultaneously, a single time point is necessarily a compromise. For determining RNA degradation rates, the optimal labeling time for a given RNA species depends on its stability, and a single labeling time point can only provide reliable estimates for a subset of transcripts with half‐lives within a specific range (Jürges et al. 2018; Uvarovskii et al. 2019). In general, shorter labeling times are optimal for more unstable RNAs. Experiments with multiple time points can provide reliable degradation rate estimates for a wider range of RNA species. *Approach‐to‐equilibrium* (Figure 4A) experiments track nucleoside analogue incorporation at various time points after labeling is initiated (Lugowski et al. 2018; Neymotin et al. 2014). With increasing labeling time, the labeled fraction for each transcript approaches an equilibrium with a rate determined by the stability of the RNA species. In *pulse‐chase* experiments (Figure 4B), another approach for measuring degradation rates, cells are pulse‐labeled with a nucleoside analogue for several hours, after which the nucleoside is withdrawn from the media, and cells are harvested at different time points (Paulsen et al. 2013; Tani et al. 2012; Uvarovskii and Dieterich 2017). RNA degradation rates can then be determined based on the decrease of the labeled fraction over time. Finally, in *pulse–pulse* experiments (Figure 4C), two different nucleoside analogues are utilized (Gasser et al. 2020; Kawata et al. 2020). After pulse labeling with nucleoside A, the culture media is replaced and nucleoside B is introduced, followed by sampling at different time points. This allows simultaneous determination of degradation and synthesis rates based on the decrease in RNAs labeled with the nucleoside A and increase in RNAs labeled with the nucleoside B, respectively. It also solves an issue of pulse‐chase experiments using one nucleoside: a cellular pool of residual or recycled nucleoside analogues can continue to produce labeled RNAs even during the chase, leading to overestimation of half‐lives (Gasser et al. 2020).

Metabolic labeling can be combined with transcriptional inhibition for measuring transcription elongation rates (Almeida et al. 2018; Calvo‐Roitberg et al. 2025; Fuchs et al. 2014; Veloso et al. 2014). Prior to metabolic labeling, cells are treated for several hours with the transcriptional inhibitor DRB (5,6‐dichloro‐1‐β‐d‐ribofuranosylbenzimidazole), which blocks transition into productive elongation but allows nascent transcripts associated with already elongating polymerases to complete transcription. Upon removal of DRB, inhibited polymerases enter productive elongation and nascent transcription from these synchronized polymerases is captured by a pulse of 4‐SU (4sUDRB‐seq; Fuchs et al. 2014), BrU (BruDRB‐seq; Veloso et al. 2014) or 5‐EU (Almeida et al. 2018). Combined with Nanopore dRNA‐seq, 4sUDRB‐seq demonstrated that more downstream transcription start sites are associated with faster elongation speeds, which leads to skipping of weaker polyadenylation sites (PASs) and use of stronger downstream PASs (Calvo‐Roitberg et al. 2025).

#### 4.2.2 Kinetics of RNA Processing

In addition to synthesis and degradation, metabolic labeling has been used to study the kinetics of RNA processing. Using exonic and intronic signals to infer gene‐level processing rates alongside synthesis and degradation rates can identify genes whose expression is limited by slow processing (de Pretis et al. 2015; Mukherjee et al. 2017; Rabani et al. 2011). However, in gene‐level analyses, the kinetics of individual processing events get convoluted, potentially masking RNA subpopulations with distinct kinetics.

Beyond gene‐level processing rates, metabolic labeling can be used to measure the splicing rates of individual introns. Splicing kinetics are generally studied using approach‐to‐equilibrium experiments with labeling times in the scale of minutes, or pulse‐chase experiments. From the labeled RNA fractions, splicing rates can be determined at the resolution of splice junctions (Mukherjee et al. 2017; Pai et al. 2017; Rabani et al. 2014), or the kinetics of the two catalytic steps of splicing can be individually dissected (Gildea et al. 2022; Wachutka et al. 2019). Metabolic labeling can also yield insights into co‐transcriptional splicing timing, as well as splicing order and coupling between splicing events in multi‐intron genes (Drexler et al. 2020). Such studies generally require long reads to simultaneously capture the splicing status of two or more introns. In nano‐COP (nanopore analysis of co‐transcriptional processing), a short 4‐SU pulse is followed by chromatin isolation and biochemical enrichment of 4‐SU RNA to ensure stringent purification of nascent RNA, which is then poly‐A tailed and sequenced by Nanopore dRNA‐seq (Drexler et al. 2020). As molecules are read from 3′ to 5′, nano‐COP reads measure RNA polymerase II position and can be used to determine splicing timing in relation to transcription. Nano‐COP revealed contrasting patterns in human and *Drosophila* splicing (Drexler et al. 2020). *Drosophila* introns were spliced more proximally to RNA pol II and their splicing generally followed the direction of transcription. While human splicing did not display such a clear directionality, neighboring introns showed coordinated splicing and terminal introns were almost exclusively spliced posttranscriptionally.

#### 4.2.3 Toward Comprehensive Modeling of the RNA Life Cycle

Modeling the RNA life cycle as consisting of synthesis, processing, and degradation still represents a highly simplified view of the eukaryotic gene expression pathway. With more elaborate study designs combining metabolic labeling, biochemical fractionation and kinetic modeling, the RNA life cycle can be further divided into various finer steps and the kinetics of each of them estimated. Two recent studies carried out 4‐SU labeling and isolated RNA from chromatin, nuclear or nucleoplasmic, and cytoplasmic fractions, as well as polysomes, followed by short‐ or long‐read sequencing (Coscujuela Tarrero et al. 2024; Ietswaart et al. 2024). Kinetic modeling was then used to estimate the rates of RNA flow between subcellular compartments (Ietswaart et al. 2024), or the rates of various mechanistic steps of the gene expression pathway in both the nucleus and the cytoplasm (Coscujuela Tarrero et al. 2024). These and other studies utilizing fine‐grained kinetic models (Müller et al. 2024; Ren, Zhou, et al. 2023; Steinbrecht et al. 2024) show that transcripts exhibit distinct combinations of kinetic rates and their expression is thus limited by different processes. For mRNAs, several recent studies agree that the majority of their lifespan is spent in the nucleus, revealing nuclear processes as a major bottleneck in the life cycle of protein‐coding genes (Ietswaart et al. 2024; Müller et al. 2024; Steinbrecht et al. 2024).

## 5 Toward Spatiotemporally Resolved RNA‐Protein Interactions

In the cell, RNAs exist as dynamic RNPs whose protein composition changes throughout the RNA life cycle and during transit between subcellular compartments. While numerous studies have catalogued the binding partners of RNA‐binding proteins (RBPs) and the protein interactomes of RNAs (Kilchert et al. 2020), in most cases these studies capture cell‐wide steady‐state snapshots of RNA‐protein interactions. On the other hand, while proximity labeling methods can be used to determine both protein and RNA composition of subcellular compartments, they provide no information on RNA–protein interactions that contribute to the regulation of RNA localization. Combining RNA labeling methods with RNA–protein interaction mapping shows promise to resolve RNA–protein interactions in time and space.

The current gold‐standard protein‐centric method for mapping RNA–protein interactions in the cell is crosslinking and immunoprecipitation (CLIP) (reviewed by Hafner et al. 2021; Xiang et al. 2024). In CLIP, RNPs are crosslinked using UV light and immunoprecipitated using an RBP‐specific antibody. RNA‐centric methods (reviewed by Gräwe et al. 2021; Kilchert et al. 2020) include RNA interactome capture (RIC), where RNPs are UV‐crosslinked, oligo‐dT beads are used to purify poly‐A RNA, and the associated proteins are identified by mass spectrometry. For details, we refer the reader to the cited reviews and limit our discussion here to methods that combine RNA–protein interaction mapping with proximity or metabolic labeling.

### 5.1 Mapping RNA–Protein Interactions in Subcellular Compartments

Combining CLIP with proximity labeling can be used to map RNA‐protein interactions with subcellular resolution. Proximity‐CLIP combines RNA metabolic labeling, protein proximity labeling and UV crosslinking to isolate RNPs localized to a specific subcellular compartment (Benhalevy et al. 2018). First, cells expressing an APEX2‐fused subcellular marker are metabolically labeled with 4‐SU to enhance efficiency of subsequent crosslinking similar to PAR‐CLIP (Hafner et al. 2010). Next, compartment‐specific biotinylation of proteins is initiated, followed by UV crosslinking of RNAs and proteins. The biotinylated and crosslinked RNPs are captured with streptavidin beads. A major limitation of Proximity‐CLIP is that it only reveals protein‐bound RNA regions, but not the identity of the bound RBPs. Two similar methods, colocalization CLIP (coCLIP) and RBProximity‐CLIP, address this limitation by introducing a two‐step purification strategy with an IP step for the RBP of interest and a streptavidin capture step to restrict the detection to a specific cellular compartment (Nowak et al. 2026; Yi et al. 2024).

### 5.2 Time‐Resolved RNA Interactomes

The combination of RNA metabolic labeling and RIC has been used to capture RNA‐protein interactions in nascent RNA and to study their dynamics. In RICK (RNA interactome using click chemistry), nascent RNA is metabolically labeled with 5‐EU followed by UV crosslinking of RNA‐protein complexes (Bao et al. 2018). The 5‐EU‐labeled RNAs are then biotinylated in fixed cells via click chemistry, the nascent RNPs are captured with streptavidin and RNA‐bound proteins identified with mass spectrometry. In a time‐resolved approach, Choi et al. performed a 4‐SU pulse‐chase with tight sampling followed by 4‐SU‐enhanced UV crosslinking, oligo‐dT capture, and mass spectrometry (Choi et al. 2024). This approach enabled tracking of the global poly‐A mRNP interactome at different stages of the RNA life cycle. While this study utilized oligo‐dT capture and was thus restricted to mRNPs that have already undergone polyadenylation, similar experiments could in principle be performed with other enrichment methods, such as antisense oligos to capture specific RNPs.

## 6 Emerging Developments and Future Outlook

Development of RNA proximity and metabolic labeling methods and concurrent advances in RNA sequencing have enabled a transition from global, static snapshots of the transcriptome to spatiotemporally resolved transcriptomics. Here, we highlight some emerging methodological developments in the field and discuss future developments. Future progress can be considered along three interconnected directions: adopting long‐read sequencing (Section 6.1.), integrating multiple spatial and temporal approaches (Section 6.2.), and extending spatiotemporal studies to increasingly complex biological systems (Section 6.3). While active development of new methods is important, there is also a need to evaluate, benchmark and improve the accessibility of existing methods. Particularly RNA proximity labeling is currently in a phase of active methods development. There are many labeling chemistries, but analyses comparing RNA profiles obtained using different chemistries are limited. While RNA metabolic labeling reagents are generally widely available, the requirement of specialized reagents that are not commercially available but require chemical synthesis (Table 1) represents a significant barrier to the wider adoption of most RNA proximity labeling methods.

### 6.1 From Short‐Read to Long‐Read Sequencing in RNA Labeling Studies

One of the more immediate developments in the field of spatiotemporal transcriptomics is the wider adoption of long‐read sequencing for RNA labeling studies. This transition shifts the focus of RNA labeling studies from genes and individual processing events to full‐length RNA molecules, allowing labeling status to be connected with the transcript isoforms, processing state, and potentially multiple regulatory events on the same RNA molecule. While long‐read sequencing is already commonly used as a readout for metabolic labeling (Coscujuela Tarrero et al. 2024; Drexler et al. 2020, 2021; Maier et al. 2020; Martinek et al. 2024), reports of proximity labeling used in conjunction with long‐read sequencing are still scarce. However, existing proximity labeling methods using affinity capture should be readily adaptable for long‐read sequencing by a change of library preparation protocol. Nanopore dRNA‐seq detection of some proximity labeling adducts, such as acylation and 8‐oxoG, might also be feasible. In support of this, acyl adducts formed by SHAPE reagents, and 8‐oxo‐dG in DNA were shown to result in discernible alterations in nanopore raw signal that enabled training of machine learning models for their detection (Aw et al. 2021; Pagès‐Gallego et al. 2025; Stephenson et al. 2022). Notably, in both proximity and metabolic labeling, the exact position of the labeled nucleotides is unimportant. Therefore, using machine learning to classify individual reads rather than nucleotides as labeled and unlabeled would be sufficient in most cases (Coscujuela Tarrero et al. 2024; Maier et al. 2020).

As discussed in Section 5, proximity labeling has been combined with CLIP for mapping RNA–protein interactions in subcellular compartments. Recently, our laboratory adapted CLIP for Nanopore direct RNA and cDNA sequencing in the dirCLIP method, enabling mapping of RBP binding sites in the context of full‐length transcripts and detection of multiple binding sites in a single RNA molecule (unpublished data). A potential future development in spatiotemporal RNA–protein interaction profiling could be combining proximity labeling and CLIP for simultaneous dRNA‐seq detection of labeling and crosslinking adducts.

### 6.2 Combining RNA Labeling Approaches

The extensive toolkit of available RNA proximity and metabolic labeling methods opens the possibility of combining multiple RNA labeling approaches in the same experiment. As discussed in Section 4, metabolic labeling with two nucleoside analogues has already been used for simultaneous measurement of synthesis and degradation rates. Similarly, combining proximity labeling methods could allow transcriptomic profiling of two cell locales in the same cell population. Finally, the combination of proximity and metabolic labeling opens the possibility of temporally resolved studies of subcellular transcriptomes. The key challenge with such approaches is to determine which chemical labels can be combined in the same experiment while avoiding perturbation of the biological system under study and enabling unambiguous detection of both labels.

Simultaneous transcriptomic profiling of two subcellular locales could be accomplished in different ways. Two affinity capture‐based methods could be combined if they utilize distinct activation mechanisms and orthogonal chemical handles. The latter point is currently the main limiting factor, as most affinity capture‐based methods rely on alkyne handles and CuAAC chemistry. To enable specific capture of labeled RNAs originating from different locales, another chemical handle utilizing a conjugation chemistry without alkyne cross‐reactivity is required. For instance, two photocatalytic proximity labeling methods using distinct wavelengths of light and installing different chemical handles could be combined. Alternatively, APEX‐seq, BAP‐seq and μMap‐seq substrates could potentially be modified to incorporate different handles, as these react directly with RNA without a secondary step (e.g., alkynylation) to introduce the chemical handle (Li et al. 2020; Liang et al. 2024; Pani et al. 2024). Mutually or sequentially orthogonal click reactions could then be used for biotin conjugation (Venrooij et al. 2024). Beyond affinity capture‐based methods, distinct labeling adducts from two proximity labeling methods could be detected by nanopore sequencing if they result in distinguishable base‐calling error profiles or raw signal alterations, as recently demonstrated for natural RNA modifications.

Combining metabolic labeling and proximity labeling could be a powerful approach for studying RNA trafficking pathways and distinguishing between transiently and more stably associated RNA subpopulations in subcellular structures. So far, studies combining the two have mainly used incorporation of electron‐rich nucleoside analogues to enhance the efficiency of APEX‐based proximity labeling or RNA‐protein crosslinking (Benhalevy et al. 2018; Li et al. 2022). However, the dynamics of nuclear speckle and Polycomb domain transcriptomes were recently studied using a combination of 4‐SU labeling and RT&Tag, a method based on in situ reverse transcription and tagmentation in specific locales determined by antibody targeting (Khyzha et al. 2025). Similar experiments could be carried out with proximity and metabolic labeling. For instance, 4‐SU labeling with nucleotide recoding could be combined with proximity labeling methods primarily labeling guanosines.

### 6.3 From Cell Lines to Single Cells, Tissues and Whole Organisms

Most RNA labeling experiments are carried out in cultured cell lines, but recent studies are expanding their applicability to fixed specimens and living organisms. While metabolic labeling requires live cells, methods for carrying out antibody or oligo hybridization‐targeted RNA proximity labeling in fixed cells are emerging (Cheng et al. 2025; Choudhury et al. 2024; Yap et al. 2025). These methods have some benefits over live‐cell methods. First, in most cases, live‐cell proximity labeling methods require either transient expression or creation of stable or genome‐edited cell lines expressing a POI fused to a labeling activity. Carrying out the labeling in fixed cells enables the use of cell lines or primary cells that are difficult to transfect or edit, as well as tissue samples. Second, although proximity labeling is generally completed in minutes, methods based on the generation of radicals can potentially induced rapid stress responses in living cells. On the other hand, use of fixed specimens raises concerns over conservation of cellular ultrastructure and RNA localization during the specimen preparation. Furthermore, with antibody‐targeted methods there are additional concerns over antibody specificity and reproducibility due to batch‐to‐batch variations in antibody preparations and future availability of commercial antibodies.

Proximity and metabolic labeling can also be used in whole living organisms. RNA proximity labeling in live animals was first demonstrated by the use of OINC‐seq in live zebrafish embryos (Lo et al. 2025). While successful in the transparent tissues of zebrafish embryos, tissue penetrance is expected to be a major limiting factor for in vivo application of light‐activated methods, although NIR light‐based methods might perform better (Li, Han, et al. 2024). BAP‐seq, where labeling is activated by the addition of a substrate for a bio‐orthogonal enzyme rather than radical generation, is a promising candidate for in vivo proximity labeling (Pani et al. 2024). In vivo RNA metabolic labeling has been utilized more extensively and used to investigate diverse processes from RNA transfer between tissues (Hunter et al. 2025) to transcriptomic responses to acute kidney injury (Yin et al. 2024) in mice. Furthermore, both endogenous and bioorthogonal metabolic enzymes have been harnessed for innovative cell type‐specific metabolic labeling approaches (Beasley et al. 2022). Finally, metabolic labeling‐based estimation of RNA synthesis and degradation rates in single cells was recently accomplished (Battich et al. 2020; Wang et al. 2025), suggesting that single‐cell and single‐nucleotide resolution RNA labeling methods will become available.

Beyond RNA labeling approaches, rapidly developing spatial transcriptomics methods offer complementary spatial information at a range of scales, often reaching single‐cell or subcellular resolution while preserving native tissue context (reviewed by Jain and Eadon 2024). Recently, imaging‐based spatial transcriptomics was integrated with RNA metabolic labeling to infer RNA kinetic parameters and localization at subcellular resolution for a targeted panel of genes (Ren, Zhou, et al. 2023). Future integration of spatial transcriptomics, RNA metabolic labeling and long‐read sequencing could enable determination of isoform‐resolved RNA dynamics and localization while preserving tissue architecture and capturing information on cellular heterogeneity and cell type differences.

## 7 Key Limitations and Open Problems

While RNA labeling methods provide a powerful toolkit to study RNA dynamics and localization, they are not without limitations. These include the potential to perturb biological processes, restricted spatial and temporal resolution, and biases and noise arising from both the labeling reaction itself and the approaches used to distinguish labeled from unlabeled RNA.

Because most RNA labeling studies are performed in living cells, a major concern is that the labeling procedure, or the genetic manipulations required to enable it, may alter the biological processes under investigation. Given the pervasive influence of naturally occurring RNA modifications on RNA metabolism, experimentally introduced modifications cannot be assumed to be biologically inert. For most RNA labeling technologies, systematic assessment of labeling‐induced toxicity, cellular stress responses, and effects on RNA metabolism remains limited. Such studies would be invaluable for defining labeling conditions that minimize perturbation while maintaining sufficient sensitivity.

Although the spatial resolution of proximity‐labeling approaches is often described in terms of a labeling radius, labeling is not confined to a sharply defined boundary but instead decreases gradually with distance from the source of labeling activity. Consequently, these methods do not provide a definitive inventory of RNAs localized to a specific cellular structure. Rather, they measure the enrichment of RNAs in the vicinity of the labeling activity, with the effective resolution influenced by multiple experimental and biological factors (Section 3). Likewise, the temporal resolution of metabolic labeling is constrained by the time required for nucleoside analogues to enter cells, undergo metabolic conversion to nucleotides, and become incorporated into RNA, as well as by the need to accumulate sufficient quantities of labeled RNA for detection.

Both proximity and metabolic labeling approaches infer the biological properties of interest indirectly. In these methods, labeling serves as a proxy for localization or RNA age, respectively, and any biases in the labeling reaction can therefore propagate into downstream analyses. For example, transcript accessibility within a subcellular compartment may influence labeling efficiency, and many labeling enzymes or chemistries exhibit sequence or nucleotide preferences that may favor specific transcripts. Differences in labeling chemistries, labeling conditions, and selection of controls may additionally influence the resulting RNA profiles. In addition, RNA labeling approaches rely on the accurate biochemical separation or computational classification of labeled and unlabeled molecules. Nonspecific carryover of unlabeled RNA and misclassification of sequencing reads can introduce substantial noise and bias.

Looking ahead, an important challenge for the field will be the development of labeling strategies that maximize sensitivity and resolution while minimizing perturbation and technical bias. Equally important will be the establishment of rigorous benchmarking frameworks and orthogonal validation approaches to enable quantitative comparisons across methods and experimental systems. Addressing these challenges will be essential for fully realizing the potential of RNA labeling technologies to investigate RNA dynamics, processing, and localization in their native cellular context.

## Author Contributions

**Antto J. Norppa:** writing – review and editing, writing – original draft, investigation, conceptualization, visualization. **Minna‐Liisa Änkö:** conceptualization, writing – review and editing, project administration, supervision, funding acquisition, resources.

## Funding

This work was supported by Sigrid Juselius Foundation (award #250273) and Jane & Aatos Erkko Foundation (grant #240018) to MLÄ.

## Conflicts of Interest

The authors declare no conflicts of interest.

## Data Availability

Data sharing is not applicable to this article as no new data were created or analyzed in this study.
